# Rev Proteins of Human and Simian Immunodeficiency Virus Enhance RNA Encapsidation

**DOI:** 10.1371/journal.ppat.0030054

**Published:** 2007-04-13

**Authors:** Sabine Brandt, Maik Blißenbach, Bastian Grewe, Rebecca Konietzny, Thomas Grunwald, Klaus Überla

**Affiliations:** Department of Molecular and Medical Virology, Ruhr-University Bochum, Germany; King's College London, United Kingdom

## Abstract

The main function attributed to the Rev proteins of immunodeficiency viruses is the shuttling of viral RNAs containing the Rev responsive element (RRE) via the CRM-1 export pathway from the nucleus to the cytoplasm. This restricts expression of structural proteins to the late phase of the lentiviral replication cycle. Using Rev-independent *gag-pol* expression plasmids of HIV-1 and simian immunodeficiency virus and lentiviral vector constructs, we have observed that HIV-1 and simian immunodeficiency virus Rev enhanced RNA encapsidation 20- to 70-fold, correlating well with the effect of Rev on vector titers. In contrast, cytoplasmic vector RNA levels were only marginally affected by Rev. Binding of Rev to the RRE or to a heterologous RNA element was required for Rev-mediated enhancement of RNA encapsidation. In addition to specific interactions of nucleocapsid with the packaging signal at the 5′ end of the genome, the Rev/RRE system provides a second mechanism contributing to preferential encapsidation of genomic lentiviral RNA.

## Author Summary

The AIDS pandemic is still an important public health problem, particularly in developing countries. A comprehensive understanding of the HIV replication cycle might allow development of new therapeutics. Despite 20 years of extensive research, the intracellular fate of the different RNAs produced during virus replication is not fully understood. It is known that the viral regulatory protein Rev binds to large viral RNAs and shuttles them from the nucleus to the cytoplasm by a cellular export pathway. We now provide evidence for a more far-reaching role of Rev. We observed that Rev enhances packaging of viral RNA into viral particles to a much larger extent than its effect on viral RNA levels in the cytoplasm. Thus, an early nuclear event (binding of Rev to the viral RNA) seems to be intimately linked to RNA encapsidation occurring at a late step of the viral replication cycle. Since Rev is not part of the viral particles, Rev seems to act indirectly, possibly by targeting the viral RNA to a cytoplasmic compartment favourable for RNA encapsidation. Thus, further studies on the function of Rev might also advance our understanding of cytoplasmic RNA trafficking and subcytoplasmic compartmentalization.

## Introduction

Virus particles of HIV-1, the major cause of the AIDS epidemic, and other members of the lentivirus family of retroviruses contain an RNA genome. After viral entry, the genomic RNA is reverse transcribed into DNA and integrates into the genome of the host cell. The integrated proviral DNA is transcribed by RNA polymerase II. Complex alternative splicing events with a major splice donor in the 5′ untranslated region (UTR) generate viral mRNAs encoding Env and a number of small regulatory proteins. The unspliced transcript serves as a template for translation of Gag and Pol proteins. During particle formation, the unspliced genomic RNA is also packaged in preference to spliced viral mRNAs and a more than 1,000-fold excess of cytoplasmic cellular RNAs. Packaging signals have been identified in the 5′UTR of lentiviruses (reviewed in [1]). To exclude encapsidation of spliced viral transcripts, packaging signals of retroviruses are generally located or extent 3′ to the major splice donor. For members of the γ-retrovirus family, a single discrete packaging motif could be identified that was necessary and sufficient for RNA encapsidation [2]. For lentiviruses, however, the situation seems to be more complex, with several RNA motifs in the 5′UTR contributing to packaging efficiency. The major packaging signal of HIV-1 seems to be a stem loop region designated SL3, the deletion of which reduces packaging efficiency approximately 20-fold [3,4]. However, transfer of the core packaging sequences from the 5′UTR to heterologous RNAs did not allow efficient packaging of the heterologous RNA [5], suggesting that additional factors are important. In HIV-2 and simian immunodeficiency virus (SIV), the major packaging signals are located upstream of the major splice donor [6–8]. It is unclear how selective encapsidation of the genomic RNA of HIV-2 and SIV over spliced viral transcripts is accomplished. As a potential mechanism, a direct interaction between nascent Gag and its RNA template has been proposed [6]. Efficient packaging and transfer of HIV-2- and SIV-based vectors by HIV-2 particles expressed *in trans* demonstrates that cotranslational packaging is not an absolute requirement. Alternatively, long-distance interactions as observed for the HIV-1 5′UTR [9] might lead to different conformations of unspliced and spliced HIV-2 and SIV transcripts favouring preferential encapsidation of the unspliced transcript.

One of the small regulatory proteins expressed from a multiply spliced transcript is the lentiviral Rev protein. Rev binds to the Rev responsive element (RRE), an RNA motif present on the unspliced transcript and the *env* mRNA (reviewed in [10]). By binding to the RRE, Rev shuttles the unspliced genomic RNA and the singly spliced *env*-mRNA via the CRM-1 export pathway from the nucleus to the cytoplasm. The unspliced genomic RNA then either serves as a template for translation of the *gag-pol* open reading frames or is packaged into the viral particle. We recently observed that omitting Rev or deleting the RRE during production of an HIV-1 vector had only a minor effect on cytoplasmic vector RNA levels, but reduced vector titers approximately 100-fold [11]. Similarly, no correlation was found between cytoplasmic RNA levels of HIV vectors containing a constitutive transport element (CTE) and vector titers [12]. Both studies indicate that cytoplasmic localization of genomic vector RNA is not sufficient for vector infectivity, and suggest that the function of the Rev/RRE system extends beyond transport of viral RNA from the nucleus to the cytoplasm. The molecular mechanism responsible for reduced titers of RRE-deficient vectors in the presence of substantial cytoplasmic vector RNA levels has not been defined. Since a Rev-independent, codon-optimized expression plasmid for HIV-1 *gag-pol* was used, the effect of Rev on vector titers could not be attributed to modulation of particle production. We therefore analyzed the effect of the Rev-RRE system on RNA encapsidation into HIV-1 and SIV particles. For both viruses, packaging efficiency of genomic vector RNA, but not cytoplasmic RNA levels, reflected the Rev effect on vector titers.

## Results

### HIV-1 Rev Enhances RNA Encapsidation

**Figure 1:**
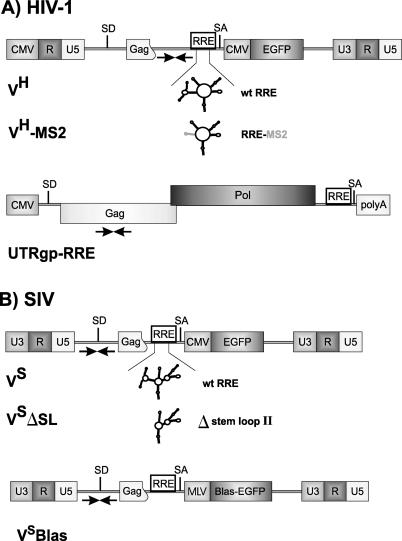
Map of HIV-1 and SIV Vector Constructs Opposing arrows mark primer binding sites for the quantitative real-time PCR. Predicted secondary structures for wild-type (wt RRE) and mutated RRE are shown, with the grey stem loop representing the MS2-derived RNA motif. (A) HIV-1 vector constructs, (B) SIV vector constructs. Blas-EGFP, EGFP cDNA fused in frame to the Blasticidin resistance gene; CMV, human cytomegalovirus immediate early promoter; EGFP, gene for the enhanced green fluorescent protein; MLV, murine leukemia virus promoter; R, direct repeat sequence; SA, splice acceptor; SD, splice donor; U3, 3′ unique sequence; U5, 5′ unique sequence.

**Figure 2:**
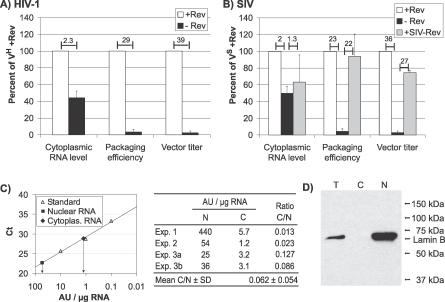
Effect of Rev on Encapsidation of HIV-1 and SIV Vector RNA (A) The HIV-1 vector plasmid V^H^ was cotransfected with expression plasmids for HIV-1 *gag-pol,* VSV-G, and HIV-1 *tat* in the presence (+Rev) or absence (−Rev) of an HIV-1 *rev* expression plasmid. Cytoplasmic RNA levels, packaging efficiency, and vector titers are presented relative to the values obtained in the same transfection experiment with V^H^ in the presence of Rev. The mean value and standard deviation of three to eight independent transfection experiments are shown. (B) The SIV vector plasmid V^S^ was cotransfected with expression plasmids for SIV *gag-pol,* VSV-G, and HIV-1 *tat* in the absence (−Rev) or presence of an SIV (+SIV-Rev) or HIV-1 *rev* (+Rev) expression plasmid. The mean value and standard deviation of four independent transfection experiments are shown. Numbers above the bars indicate fold induction by Rev. (C) Using a real-time PCR for preGAPDH RNA, threshold cycle numbers (Ct) were determined for four nuclear (N) and cytoplasmic (C) RNA preparations in three independent experiments. Relative amounts of preGAPDH RNA levels were calculated as arbitrary units (AU) per microgram extracted RNA by including 10-fold serial dilutions of standard nuclear RNA preparations. Ct values of a typical experiment are shown in the left panel, and the table summarizes the results. (D) Western blot analysis of total cell lysate (T), and cytoplasmic (C) and nuclear (N) fractions for Lamin B.

Infectious HIV-1-based vector particles were produced by cotransfection of a codon-optimized HIV-1 *gag-pol* expression plasmid, a VSV-G expression plasmid, a *tat* expression plasmid, and the prototypic HIV-1 vector construct V^H^ (Figure 1A) into 293T cells in the presence or absence of a *rev* expression plasmid [11]. Vector titers in the absence of Rev were only 3% of those obtained with Rev (Figure 2A). To study the role of Rev on packaging efficiency, we determined genomic vector RNA levels in the cytoplasm of transfected cells and in viral particles pelleted from the supernatant of transfected cells through a 30% sucrose cushion. Mean cytoplasmic V^H^ vector RNA copy numbers per microgram extracted RNA were 1.7 × 10^9^ ± 1.1 × 10^9^ and 7.1 × 10^8^ ± 3.5 × 10^8^ in the presence and absence of Rev, respectively, matching well with results obtained in Northern blot analyses (unpublished data). To exclude the possibility that Rev-independent cytoplasmic localization of vector RNA is simply due to contamination of the cytoplasmic RNA fraction with large amounts of nuclear RNA, pre–glyceraldehyde-3-phosphate dehydrogenase (GAPDH) RNA levels in cytoplasmic and nuclear RNA preparations were determined by quantitative real-time PCR. As expected for a nuclear RNA, 16-fold higher concentrations of preGAPDH RNA were observed in the nuclear fraction than in the cytoplasm (Figure 2C). In addition, Western blot analysis revealed the presence of lamin B in the nuclear fraction, but not in the cytoplasmic fraction, confirming the fidelity of the cell fractionation procedure (Figure 2D).

Mean V^H^ vector RNA copy numbers in particles were 5.4 × 10^8^ ± 2.0 × 10^8^ per milliliter supernatant of transfected cells with Rev, and 6.1 × 10^6^ ± 4.6 × 10^6^ per milliliter without Rev. To normalize encapsidation for cytoplasmic RNA levels, the ratio of particle-associated RNA copies per milliliter supernatant and cytoplasmic RNA copies per microgram extracted RNA was taken as packaging efficiency. The ratio obtained for V^H^ in the presence of Rev was set as 100%, and the values of all other samples are given relative to this (Figure 2A). Omitting Rev reduced the packaging efficiency to 3%, while cytoplasmic RNA levels were only reduced to 44% of those obtained in the presence of Rev. Thus, Rev enhances packaging efficiency and titer of the HIV-1 vector to a similar degree.

### Rev Enhances RNA Encapsidation into SIV Particles

Analogous experiments were also performed with an SIV vector packaging system [13] to determine whether the enhancing effect of Rev on vector RNA packaging is conserved among primate lentiviruses. Since HIV-1 Rev and Tat can functionally replace the SIV homologues, SIV vector particles were prepared by cotransfection of expression plasmids for codon-optimized *gag-pol* of SIVmac239, VSV-G, and HIV-1 *tat* with a prototypic SIV vector construct (V^S^ in Figure 1B) in the presence and absence of the HIV-1 *rev* expression plasmid. The same enhancing effect of HIV-1 Rev on SIV vector titer and vector RNA packaging efficiency (Figure 2B) was observed, while cytoplasmic RNA levels of the SIV vector were only reduced 2-fold in the absence of Rev. To analyse whether SIV Rev also enhances RNA encapsidation, an SIV *rev* expression plasmid was cotransfected with expression plasmids for SIV *gag-pol,* VSV-G, and HIV-1 *tat*. Similar to Rev of HIV-1, SIV Rev also enhanced SIV vector RNA packaging efficiency and titer of the SIV vector (Figure 2B).

### Rev-Independent Formation of Viral Particles

**Figure 3:**
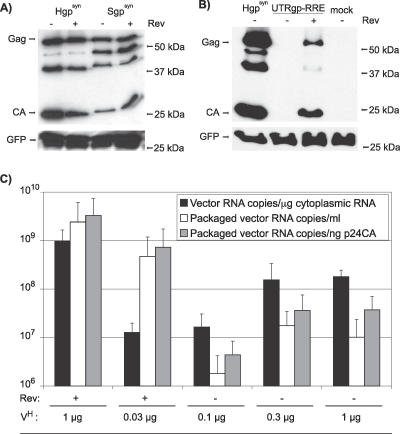
Effect of Rev on Gag-Pol Particle Formation Viral particles were pelleted from the supernatant of cells transfected in the presence (+) or absence (-) of an HIV-1 *rev* expression plasmid with codon-optimized *gag-pol* expression plasmids of SIV (Sgp^syn^) and HIV-1 (Hgp^syn^) (A) or a wild-type HIV-1 *gag-pol* expression plasmid (UTRgp-RRE) (B). Western blot analyses were performed with pelleted particles and a CA-specific monoclonal antibody (upper panel). To control for transfection efficiency, a GFP expression plasmid had been included during transfection, and lysates of transfected cells were analyzed using an anti-GFP antibody (lower panel). (C) Cytoplasmic and particle-associated vector RNA copy numbers were determined in cell cultures transfected with different amounts of the V^H^ vector plasmid in the presence or absence of Rev expression plasmid. In addition, pelletable p24CA levels were determined and used to calculate the packaging efficiency as particle-associated vector RNA copies per nanogram p24CA.

The enhanced packaging efficiency we observed in the presence of Rev could be due to stimulation of particle production and release. It has been previously observed that Rev enhances Gag protein levels in a RRE-dependent manner to a larger extent than its cytoplasmic mRNA levels [14]. Although this seemed unlikely under our experimental conditions, since codon-optimized *gag-pol* expression plasmids lacking RRE and 5′UTRs were used, we cotransfected the *gag-pol* expression plasmids of HIV-1 and SIV in the presence and absence of the HIV-1 *rev* expression plasmid. Particles released in the supernatant were pelleted, and a Western blot analysis with an anti-HIV-1 p24 capsid (CA) antibody also cross-reacting with SIV CA was performed. Rev did not affect the amounts of particles produced by the codon-optimized expression plasmids, and no obvious differences in the processing of Gag could be observed (Figure 3A). In contrast, expression of wild-type *gag-pol* from an expression plasmid also containing the HIV-1 RRE (UTRgp-RRE, Figure 1A) could only be detected in the presence of Rev (Figure 3B). The large difference at the protein level in UTRgp-RRE-transfected cultures was not reflected in cytoplasmic *gag-pol* RNA levels, which were reduced approximately 5-fold in the absence of Rev (unpublished data).

### Relationship of Cytoplasmic RNA Levels and Packaging Efficiency

The slightly reduced cytoplasmic RNA levels observed in the absence of Rev could lead to large differences in RNA packaging due to a non-linear relationship. Therefore, increasing amounts of the V^H^ vector plasmid were cotransfected with constant amounts of packaging plasmids in the absence of Rev and compared with transfections containing a small amount of V^H^ in the presence of Rev. Cytoplasmic RNA levels obtained with large amounts of V^H^ in the absence of Rev were up to 10-fold higher than the levels obtained with a low amount of V^H^ in the presence of Rev (Figure 3C). Despite much lower cytoplasmic RNA levels, particle-associated vector RNA copies per milliliter supernatant of cells transfected with V^H^ in the presence of Rev exceeded those obtained in the absence of Rev. Thus, a non-linear relationship between cytoplasmic RNA levels and packaging efficiency can not explain the differences in packaging efficiency observed.

Instead of measuring packaging efficiency as the ratio of cytoplasmic RNA to particle-associated RNA, the packaging efficiency is frequently determined as amount of vector RNA per amount of CA. Therefore, pelletable p24 CA levels were also determined in the supernatant of the transfected cells. Neither Rev nor the amount of transfected vector plasmid influenced p24 CA levels notably. However, the amount of particle-associated RNA per microgram p24CA was strongly reduced in the absence of Rev, confirming the influence of Rev on RNA packaging (Figure 3C).

### Effect of Rev on RNA Encapsidation in Infected Cells

**Figure 4:**
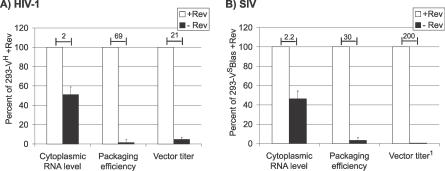
Effect of Rev on RNA Packaging in Cells Infected with HIV-1- or SIV-Based Vectors (A) HIV-1-based vectors. 293T cells stably infected with the V^H^ vector were transfected with Hgp^syn^ and expression plasmids for VSV-G and HIV-1 *tat* in the presence (+Rev) or absence (−Rev) of the HIV-1 *rev* expression plasmid. (B) SIV-based vectors. 293T cells stably infected with V^S^-Blas were cotransfected with Sgp^syn^ and VSV-G and *tat* expression plasmids in the presence or absence of the *rev* expression plasmid. Cytoplasmic RNA levels, packaging efficiencies, and vector titers are presented relative to those obtained in the same transfection experiment in the presence of Rev. The mean value and standard deviation of three independent transfection experiments are shown. Numbers above the bars indicate fold induction by Rev. ^1^, result of single transfection experiment.

The minor effect of Rev on cytoplasmic vector RNA levels could be due to overexpression of the vector RNA by transient transfection. To study the role of Rev on RNA encapsidation under more natural expression levels, 293T cells were infected with HIV-1 vector particles transferring the V^H^ vector, and a stable cell line, designated 293-V^H^, was established. The V^H^ vector could be rescued from these cells at a titer of 5.8 × 10^4^ GFU/ml by transient cotransfection of expression plasmids for HIV-1 *gag-pol,* VSV-G, *tat,* and *rev*. Mean cytoplasmic vector RNA copy numbers per microgram extracted RNA were 1 × 10^7^ ± 4.3 × 10^6^, and thus approximately 100-fold lower than after transient transfection of the vector construct. Even at these reduced expression levels, transfection of the *rev* expression plasmid only led to a 2-fold increase in cytoplasmic RNA levels (Figure 4A). In contrast, Rev enhanced packaging efficiency and the vector titer 69- and 21-fold, respectively. Similar results were obtained in cells stably infected with an SIV vector (Figure 4B).

### Analysis of RRE Deletion Mutants

**Figure 5:**
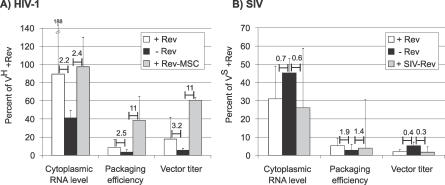
Relevance of the Rev–RRE Interaction (A) An HIV-1 vector plasmid in which stem loop II of the RRE was replaced by the MS2 stem loop was cotransfected with expression plasmids for HIV-1 *gag-pol,* VSV-G, and *tat* in the absence (−Rev) or presence of a *rev* (+Rev) expression plasmid, or in the presence of an expression plasmid encoding a fusion protein of Rev and the MS2 coat protein (+Rev-MSC). (B) An SIV vector with a deletion (V^S^ΔSL in Figure 1B) of stem loop II of the RRE was cotransfected with expression plasmids for SIV *gag-pol,* VSV-G, and *tat* in the absence (−Rev) or presence of an HIV-1 (+Rev) or SIV *rev* expression plasmid. Cytoplasmic RNA levels, packaging efficiency, and vector titers were determined as described, and all values are expressed relative to the values obtained in the same transfection experiment for the parental V^H^ (A) and V^S^ (B) vectors in the presence of Rev, which were set as 100%. Mean value and standard deviation of at least three independent transfection experiments are shown. Numbers above the short vertical lines indicate fold induction by Rev.

To determine whether the newly discovered function of Rev was dependent on interaction with the RRE, stem loop II of the RRE, which is essential for Rev binding, was replaced by a heterologous stem loop from the bacteriophage MS2 in the context of the V^H^ vector, resulting in V^H^-MS2 (Fig 1A). Packaging efficiency and vector titers of V^H^-MS2 in the presence of Rev were reduced to 9% and 18% of the values obtained with V^H^, respectively (Figure 5A). Despite deletion of stem loop II of the RRE, Rev enhanced the packaging efficiency and titer of the HIV-1 V^H^-MS2 vector by 2.5- and 3.2-fold, respectively (Figure 5A). This minor effect of Rev could either be due to residual binding of Rev to the remaining RRE, or to a second Rev-binding site recently identified in the 5′UTR of HIV-1 [15,16]. Using an SIV vector containing a deletion of stem loop II of the SIV RRE (V^S^ΔSL in Figure 1B), HIV-1 and SIV Rev did not enhance packaging efficiency and vector titer either (Figure 5B).

### Tethering of Rev to the Vector RNA via a Heterologous RNA Element

Stem loop II of the RRE of the V^H^-MS2 vector had been replaced by an MS2 stem loop, which is the binding site for the coat protein of the MS2 bacteriophage. Fusion of the Rev protein to the MS2 coat protein allowed tethering of Rev to the MS2 stem loop–containing RNAs independent of the RRE [17,18]. Cotransfection of an expression plasmid for a fusion protein of Rev and the MS2 coat protein (Rev-MSC) with V^H^-MS2 enhanced packaging efficiency and titer of the V^H^-MS2 vector 11-fold (Figure 5A). Thus, Rev-mediated enhancement of encapsidation efficiency largely depends on binding of Rev to the RRE.

## Discussion

The main function attributed to the Rev proteins of immunodeficiency viruses is the shuttling of viral RNAs containing the RRE from the nucleus to the cytoplasm, allowing expression of viral structural genes [10]. The magnitude with which Rev enhanced export of RRE-containing RNAs varied greatly with experimental conditions reaching from virtually no effect of Rev on cytoplasmic RNA levels [19] over approximately 10-fold effects [14,20,21], to an absolute requirement for Rev [22]. A suboptimal level of splicing is considered to be crucial to reveal the full nuclear export function of Rev [10]. If splicing is too efficient, the RRE-containing intron is removed, and potential target mRNAs of Rev can not accumulate in the nucleus. Inefficient splicing due to a lack of recognition by splicing factors and insufficient retention in the nucleus might lead to constitutive export independent of Rev. In addition, the presence of *cis*-acting repressive sequences and/or instability sequences in the viral genome [23–26] most likely also influences nuclear retention and thus the degree of Rev responsiveness. However, even if comparable proviral constructs with mutations in Rev or RRE were used, the Rev effect on cytoplasmic RNA levels varied. Transient transfection experiments mostly revealed an approximately 5- to 10-fold enhancement of cytoplasmic expression levels of Rev-dependent viral RNAs [14,20,21]. In contrast, in a lymphoid cell line stably infected with a Rev-negative HIV-1, Rev-dependent RNAs could not be detected in the cytoplasm, demonstrating an absolute requirement for Rev under these experimental conditions [22]. While the transient transfection experiments could be questioned due to overexpression, a selection bias can not be formally excluded in the stably infected lymphoid cell lines. Consistent with an enhanced polysomal association of RRE-containing RNAs [20], Rev was also found to stimulate protein expression levels to a greater extent than the cytoplasmic mRNA levels encoding this protein [14].

We then observed another function of Rev, the enhancement of RNA encapsidation. Since Rev enhances cytoplasmic levels of viral genomic RNA and *gag-pol* expression, it is difficult to analyse the effect of Rev on steps of the viral replication cycle that depend on cytoplasmic RNA and Gag-Pol protein levels, particularly in virus-infected cells or in cells transfected with proviral expression plasmids. The use of lentiviral vector systems with codon-optimized *gag-pol* expression plasmids allowed us to establish experimental conditions in which *gag-pol* expression is completely independent of the Rev/RRE system [13]. In addition, removal of large parts of the viral genome, including *cis*-acting repressive sequences and/or instability sequences [23–26] from the vector construct, led to a vector RNA, the cytoplasmic expression of which was largely independent of the Rev/RRE system. Under these conditions, the influence of Rev on RNA export and expression could be avoided, and the enhancing effect of Rev on RNA encapsidation became clearly evident.

Mechanistically, enhancement of RNA encapsidation by Rev was dependent on Rev binding the vector RNA to be packaged. A functional RRE was not required, as tethering of Rev to the vector RNA via a heterologous stem loop also enhanced packaging. Since Rev is not part of the viral particle, the question of how Rev enhances RNA encapsidation arises. One attractive hypothesis is that binding of Rev to the RRE in the nucleus shuttles the viral genomic RNA via the CRM-1 export pathway to a subcytoplasmic compartment, facilitating subsequent sequence-specific interactions of the nucleocapsid of Gag with the packaging signals in the 5′UTR of the genomic RNA. Colocalization and fluorescence resonance energy transfer studies suggest a perinuclear localization for this meeting point [27]. A coupling of nuclear export and RNA encapsidation would also explain why the major packaging signals of HIV-1 are not sufficient for efficient packaging [5,28]. Although a 1.1-kb *env* gene fragment containing the RRE enhanced packaging in initial experiments with a vector containing an extended *gag* sequence [28], deletion of the RRE from the *env* fragment in a subsequent study with vectors containing only the first 43 nucleotides of *gag* reduced encapsidation levels just 2-fold [29]. Insertion of a similar *env* fragment into a heterologous RNA containing a fragment spanning most of HIV-1 5′UTR and a CTE from Mason-Pfizer monkey virus at the 3′ end did not lead to detectable packaging of the transcript [5]. In both of the latter publications, the RNA to be packaged might have been efficiently transported into the cytoplasm via the TAP-dependent mRNA export pathway [30], preventing Rev-induced export via the CRM-1 pathway even in the presence of Rev. Despite wild-type levels of cytoplasmic RNA of RRE-deficient, CTE-containing vectors, titers were severely impaired [12]. This would suggest that export via the CRM-1 pathway is necessary for efficient packaging of the genomic RNA of lentiviruses. However, substitution of the RRE of HIV-1 by the CTE in the context of proviral expression plasmids led to efficient packaging [31]. Under these experimental conditions, nuclear export of both the *gag-pol* encoding RNA and the genomic RNA to be packaged are dependent on the CTE. Therefore, it seems that the packaging efficiency can also be influenced by the export pathway of the *gag-pol* encoding RNA. This is further supported by the observation that subcellular localization of Gag and HIV-1 virion assembly in murine cells greatly depends on the export pathway of the encoding mRNA [32]. Export of the *gag-pol* encoding RNA and the genomic RNA to be packaged by the same specialized export pathway might lead to enrichment of nascent Gag protein and packageable RNA in a subcytoplasmic compartment facilitating encapsidation. Although an influence of the nuclear export pathway of the *gag* encoding RNA on packaging efficiency and/or vector titers would reconcile results from the literature, this hypothesis needs to be addressed directly. At present, alternative hypotheses, in which Rev tethers the viral genomic RNA to Gag via bridging cellular proteins prior to dissociation of Rev from the RRE independent of the nuclear export function, can not be excluded either.

Whatever the precise mode of action, the magnitude of the effect of Rev on packaging is comparable to or exceeds the effect of deletions of parts of the multipartite HIV-1 packaging signal. Deletion of SL3, the dominant packaging domain in the 5′UTR of HIV-1, reduces RNA encapsidation approximately 20-fold [3,4], while Rev enhanced RNA packaging 23- to 69-fold. Thus, in addition to interaction of the nucleocapsid of Gag with the packaging signal at the 5′ end of the genome, the Rev/RRE system provides lentiviruses with a second mechanism contributing to preferential encapsidation of genomic RNA.

## Materials and Methods

### Plasmids.

The HIV-1 vector constructs HIV-CLCG [33] and HIV-CLCG-MS [11] were modified by insertion of nucleotide sequences 1115 to 1329 of SIVmac239 (numbering according to GenBank entry M33262) in antisense orientation into the blunt-ended EcoN1 site of the vectors generating V^H^ and V^H^-MS2, respectively. V^S^ (VCGΔSB in reference [34]) contains nucleotide sequences 1–2030, 8015–9499, 9689–9731, and 10057–10535 of the proviral SIVmac239 clone. Stem loop II (nucleotides 8482–8548) of the SIV-RRE was deleted by overlap extension PCR, resulting in V^S^ΔSL. The green fluorescent protein (GFP) Blasticidin fusion gene expressing vector V^S^Blas was constructed by deleting nucleotides 8899–9375 and all remaining nucleotides between 2031 and 8014 from VGBlasΔBH [35].

The expression plasmids encoding VSV-G (pHIT-G), HIV-1 *tat* (pcTat), HIV-1 *rev* (pcRev), a fusion protein of HIV-1 Rev and capsid of bacteriophage MS2 (Rev-MSC), and codon-optimized *gag-pol* of HIV-1 and SIV, have been described previously [13,17,36,37]. An 840-bp fragment encompassing the RRE of HIV-1 was inserted into the Xho-I site of pcUTRgp [13] to generate UTRgp-RRE.

### Production and characterization of vector particles.

Vector-containing particles were produced by transfection of 293T cells using the calcium phosphate coprecipitation method as described [38]. Two days after transfection, the supernatant of the transfected cells was passed through a 0.45-μm filter and stored at −80 °C or used directly for titration on 293 cells [39]. Viral particles were also pelleted through a 30% sucrose cushion by ultracentrifugation and analyzed by Western blot with the HIV-1 p24-specific antibody 183-H12-5C (US National Institutes of Health [NIH] AIDS Research and Reference Reagent Program, http://www.aidsreagent.org) directed against HIV-1 and SIV capsid. The amount of pelleted p24CA was determined using the INNOTEST HIV Antigen mAb Kit (Innogenetics, http://www.innogenetics.com). To control for transfection efficiency, GFP expression was determined in lysates of transfected cells using an anti-GFP antibody (Santa Cruz Biotechnology, http://www.scbt.com). For establishment of stable cell lines 293-V^H^ or 293-V^S^Blas, vector-containing particles were produced as described above. 293T cells were transduced with V^H^ vector particles on three consecutive days. Cells were then sorted for GFP expression by flow cytometry, resulting in a population of 78% GFP-positive cells. For 293-V^S^Blas cells, 293T-cells were transduced once and selected for Blasticidin resistance essentially as described [35].

### Packaging assay.

To prepare cytoplasmic RNA, trypsinized cells were washed with PBS, and the plasma membrane was permeabilized in buffer RLN (50 mM Tris-Cl, 140 mM NaCl, 1.5 mM MgCl_2_, 0.5% Nonidet P-40, 1,000 U/ml RNase inhibitor, 1 mM DTT) for 5 min on ice. Intact nuclei were pelleted by centrifugation for 2 min at 300*g* at 4 °C. The supernatant was harvested as cytoplasmic fraction from which the RNA was isolated with the RNeasy Mini Kit (Qiagen, http://www.qiagen.com). Viral particles pelleted as described above were resuspended in 140 μl of PBS, and RNA was extracted using the QIAmp Viral RNA Mini Kit (Qiagen).

Cytoplasmic and particle-associated RNA levels of V^H^, V^S^, and their derivatives were determined by quantitative real-time PCR as described previously [39]. This PCR detects a 215-bp fragment spanning the major splice donor of SIVmac239. To allow detection of genomic HIV vector RNAs with the same PCR, the SIV PCR fragment had been introduced into the HIV-1 vector constructs in antisense orientation downstream of the truncated *gag* gene. Omitting the reverse transcriptase from the RT-PCR was used to confirm elimination of transfected plasmid DNA by DNAse treatment of extracted RNA samples.

To validate the cellular fractionation procedure, cytoplasmic RNA was extracted as described above. Nuclear RNA was also extracted from the pelleted nuclei after one washing step in PBS. Then, 500 ng of cytoplasmic and nuclear RNA were analyzed by quantitative real-time PCR with the primers preGAP-DHE6s and preGAP-DHE6a amplifying intronic sequences of the preGAPDH RNA [39]. Serial dilutions of nuclear RNA served as standards and allowed us to determine the ratio of cytoplasmic to nuclear preGAPDH RNA levels. In addition, the protein content of the nuclear pellet (prepared as described above and resuspended in lysis buffer [50 mM Tris-HCl (pH 7.4); 150 mM NaCl; 40 mM NaF; 5 mM EDTA; 5 mM EGTA; 1% (v/v) Nonidet P-40; 0.1% (w/v) Natriumdesoxycholat; 0.1% (w/v) SDS]) and the cytoplasmic fraction was determined using the Bio-Rad protein assay (Bio-Rad, http://www.bio-rad.com). Next, 40 μg protein of each fraction were analyzed by Western blot using the anti-Lamin B antibody C-20 (Santa Cruz Biotechnology). A total cell lysate was also included.

A heterologous RNA transiently expressed at comparable levels in the cytoplasm was packaged approximately 500-fold less efficient as the vector RNAs, confirming the specificity of the packaging assay [39]. Cytoplasmic RNA levels of UTRgp-RRE were determined by an HIV-1 *gag*-specific RT-PCR using primers SK145 and SKCC1B from the Amplicor HIV-1 Monitor test [40].

## Supporting Information

### Accession Numbers

The GenBank (http://www.ncbi.nlm.nih.gov/Genbank) accession numbers for the genes and gene products discussed in this paper are HIV-1 vector pNL4–3 (AF324493), human GAPDH (J04038), and simian (macaque) immunodeficiency virus, isolate 239 (M33262).

## Abbreviations

CA: capsid
CTE: constitutive transport element
GAPDH: glyceraldehyde-3-phosphate dehydrogenase
GFP: green fluorescent protein
RRE: Rev responsive element
SIV: simian immunodeficiency virus
UTR: untranslated region
